# OxyContin reformulation and drug-related-arrest rates, property-related crimes, child maltreatment and food pantry participation

**DOI:** 10.3389/fphar.2026.1681241

**Published:** 2026-03-27

**Authors:** Hang Zhao, Xiaohan Sun

**Affiliations:** 1 The Business and Economics Department, The Computer and Information Science Department, Allegheny College, Meadville, PA, United States; 2 The Business and Economics Department, Allegheny College, Meadville, PA, United States

**Keywords:** crimes, drug abuse, food pantry participation, OxyContin, violations

## Abstract

**Background:**

Many policies at the federal and state level have been implemented to curb the opioid crisis. A signature policy, the OxyContin reformulation, was introduced in 2010. However, an increasing body of research has documented negative impacts of the OxyContin reformulation, such as a rise in heroin use and overdose deaths.

**Objective:**

This study evaluates other unintended impacts of the OxyContin reformulation on the arrest rates for drug abuse violations, the arrest rates for drug offenses, the rates for property related crimes, child maltreatment and food pantry participation rate.

**Methods:**

A difference-in-differences framework, event study specifications, and a state-year-level representative longitudinal sample to exploit cross-state variation in the OxyContin misuse rate prior to the reformulation. All specifications include state and year fixed effects, as well as state-level time-varying covariates, including demographic composition (e.g., population shares ages 0–19, 20–39, and 65+) and relevant state policy indicators.

**Results:**

We find evidence consistent with a positive causal relationship between the OxyContin reformulation, arrest rates for drug abuse violations, and rates for motor vehicle theft.

**Conclusion:**

Findings indicate that future drug policies should more carefully consider the various needs of opioid users and potential negative externalities following the implementation of new policies.

## 1 Introduction

Drug overdose deaths in the United States increased by approximately 140% between 2000 and 2014 (Rudd et al., 2016; Alpert et al., 2018), and the rate rose by 14% per year from 2013 through 2016 (Hedegaard et al., 2020). Drug overdoses have reached epidemic levels over the past 20 years (Paulozzi, 2012). The United States is experiencing its worst overdose crisis, primarily driven by the overprescription of opioid pain medications (Kolodny et al., 2015; Beheshti, 2019; Rummans et al., 2018; Makary et al., 2017). Opioid-related overdose deaths have risen rapidly by 200% since 2000, according to the Centers for Disease Control and Prevention (CDC) (Stevens et al., 2017). Thus, the opioid crisis and related negative consequences urgently need to be addressed through targeted policies, such as the OxyContin reformulation.

To curb prescription opioid abuse from the supply side, the federal government and states have implemented programs such as prescription drug monitoring programs (PDMPs). In addition, the Food and Drug Administration (FDA) has approved abuse-deterrent versions of several opioid brand names; these include Targiniq, Embeda, Hysingla, MorphaBond, Xtampza, and Troxyca (Alpert et al., 2018). Also, an abuse-deterrent version of OxyContin, the most widely abused prescription opioid, was introduced by Purdue Pharma and approved by the FDA in 2010.

The abuse-deterrent version was designed to render the pill hard to break, crush, or dissolve; in contrast, the original formulation was easy to abuse by chewing, snorting, or injecting a crushed pill (Alpert et al., 2018; Butler et al., 2013; Mackenzie-Liu, 2021). When the OxyContin reformulation replaced the original version, OxyContin abuse decreased by 35% (Cicero et al., 2012). However, a growing literature has found that the reformulation also had negative impacts; for instance, heroin use and overdose deaths increased as individuals who previously misused OxyContin turned to heroin, marking the second wave of the opioid crisis (Alpert et al., 2018; Beheshti, 2019; Evans et al., 2019).

**FIGURE 1:**
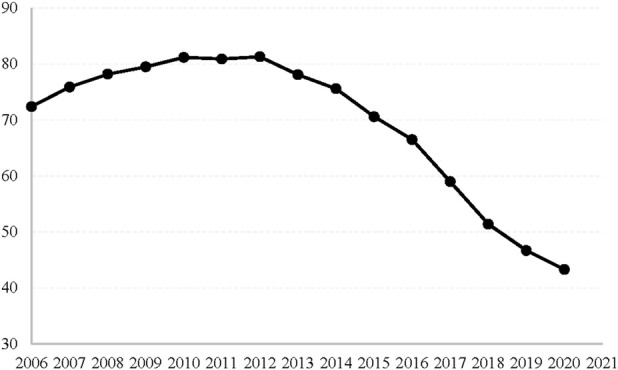
U.S. State opioid dispensing Rate per 100 persons from 2006 to 2020. Source: centers for disease control and prevention, national center for injury Prevention and control.

Figure 1 shows the U.S. state opioid dispensing rates per 100 persons from 2006 to 2020. We can find that the overall national opioid dispensing rate declined from 2012 to 2020. Thus, the reformulation could ultimately achieve the purpose of reducing prescription opioid abuse and overdose deaths. However, under the Iron Law of Prohibition (Cozzi, 2007), more compact substitutes, such as heroin, reduces the intended benefits of reformulation and may lead to unintended consequences. For instance, Maclean et al. (2021) found that despite policy interventions, an average of 128 Americans has died from opioid overdoses every day since 2018, with the total economic cost of opioid abuse is estimated to exceed $500 billion annually. Drug-related crimes have cost the US criminal justice system $167 billion (Cozzi, 2007). Studying the impact of the OxyContin reformulation as a sizeable national supply intervention and the associated increases in drug-related crimes will help us understand whether the reformulation serves the original purpose and has adverse effects on drug abuse violations and property-related crimes, and whether it imposes an additional economic burden.

The opioid crisis began in the mid-1990s and costs at least $596 billion in the U.S. annually (Szalavitz and Rigg, 2017). Following Maclean et al.’s (2022) perspective, the crisis can be separated into three waves: Mid-1990s through 2010, 2010-2013 and 2013 and onward. We are currently in the third decade, and it began with significant increases in overdose deaths involving synthetic opioids, such as fentanyl (Gladden et al., 2016; O’Donnell et al., 2017a; O’Donnell et al., 2017b). Fentanyl has heroin-like effects and can be combined with heroin to increase its euphoric effects. Focusing on the second wave of the opioid crisis, this study estimates the potential indirect impact of OxyContin’s reformulation on the arrest rate for drug abuse violations, a term used by the Federal Bureau of Investigation’s (FBI) Uniform Crime Reporting (UCR) Program to classify arrests related to the unlawful possession, sale, use, cultivation, and manufacture of narcotics (Yamamoto and Ran, 2013; U.S. Department of Justice, 2004). Our findings provide insights for policymakers addressing the ongoing third wave.

The UCR Program further categorizes these arrests based on the type of drug involved, such as opium, heroin, marijuana, methadone, and barbiturates (Yamamoto and Ran, 2013; U.S. Department of Justice, 2004). While we retain the term “drug abuse violations” for consistency with the official UCR classification, we acknowledge that it may be considered outdated or stigmatizing. Throughout this paper, we use more neutral terms such as “drug-related arrests” or “drug-related offenses” where appropriate, in line with current best practices in public health research. Data are reported by thousands of law enforcement agencies, including federal and local/state law enforcement agencies without overlapping jurisdictions (U.S. Department of Justice, 2004). According to the FBI’s Crime Data Explorer, drug offenses are always the highest arrest category in the United States.

Following the reformulation of OxyContin, which made it harder to misuse, many individuals who had previously misused the drug began using heroin, a potent and illicit opioid (Alpert et al., 2018; Evans et al., 2019). Heroin is generally cheaper and more readily available than prescription opioids, making it an attractive substitute for individuals with opioid dependence (Abuse, 2018). However, given heroin’s high addictiveness, continued substance use and ongoing dependence can increase overall drug expenditures, which may in turn lead some individuals to engage in property crimes such as motor vehicle theft to finance continued drug use. This behavioral pattern is consistent with prior research showing a strong association between drug use and criminal activity, particularly property crime in low-income populations (Harrison and Gfroerer, 1992).

The assumption is informed by prior research linking illicit drug use to increases in crime. For example, Rosenfeld et al. (2017) found that since 2013, drug-related homicides have increased more than other types of homicide (Rosenfeld et al., 2017). Other studies find that increases in heroin use is positively associated with homicide, other violent crimes, and property crimes at different geographic levels (Szalavitz and Rigg, 2017; Anglin and Speckart, 1988; Barton, 1980; Nurco et al., 1988; Park, 2021; Johnson et al., 1985).

At the same time, the existing literature provides mixed evidence regarding the behavioral consequences of abuse-deterrent reformulations. For instance, Alpert et al. (2018) find evidence consistent with substitution from prescription opioids to heroin following the reformulation of OxyContin (Alpert et al., 2018), while Wolff et al. (2020) find no significant effect of OxyContin’s reformulation on heroin initiation or prescription pain reliever misuse (Wolff et al., 2020). In addition, at least one study reports no association between heroin use and violent crime, which may reflect the sedative properties of heroin that reduce aggressive behavior (Szalavitz and Rigg, 2017). Overall, these findings suggest that the relationship between opioid reformulation, substance use, and crime remains empirically ambiguous.

According to the National Institute on Drug Abuse (NIDA), individuals who use illicit drugs are more likely to engage in criminal behavior, especially while under the influence (Abuse, 2021). Thus, arrests for drug abuse violations can elucidate the link between the rise in the use of illicit substitutes for OxyContin, such as heroin, and other crimes, such as burglary, larceny and motor vehicle theft. By investigating the effects of the OxyContin reformulation on drug abuse violations, this study can, to some extent, account for other crimes. Of course, some successful law enforcement in the early 2000s might hurt the independent causality of the reformulation and bias the results. To provide a credible investigation, a well-designed identification strategy and multiple falsification tests will be conducted in the study.

Due to the increase in demand substitution, the illicit drug heroin may be responsible for a more significant portion of offenses, which could hurt public security. By investigating whether the OxyContin reformulation causes higher arrest rates for drug-related violations and offenses and rates for property crimes, burglary, larceny, and motor vehicle theft, the results of this study will provide evidence for policymakers, government agencies, and organizations seeking to develop more comprehensive and innovative public policy. Failure to address those unintended negative impacts when developing policies would do more harm than good. Policymakers should realize that OxyContin abusers are part of the community; thus, creating a comprehensive and innovative approach should adequately consider their needs and the associated assistance.

## 2 Data and measures

This study uses a range of state-year-level data sources spanning 2001 to 2019. The primary outcome variables include the number of arrests for drug abuse violations and drug offenses at the state level, derived from the Federal Bureau of Investigation’s Crime Data Explorer for 2001-2019. Arrest data were collected from the Summary Reporting System (SRS) and National Incident-Based Reporting System (NIBRS) reports submitted to the FBI (Arrests Offense Counts, 2019). The arrests for drug abuse violations variable includes behaviors such as the unlawful possession, sale, use, cultivation, and manufacture of narcotic drugs, including opium, heroin, marijuana, methadone, and barbiturates (Yamamoto and Ran, 2013; U.S. Department of Justice, 2004). In addition, we combined arrests for drug possession and drug sales as a variable for arrests for drug offenses (Butler et al., 2013). The fraction of total arrests divided by state population was used to measure the arrest rates for drug abuse violations and drug offenses for each state from 2001 to 2019.

Property crimes, burglary, larceny, motor vehicle theft, and violent crimes were measured using data from the Summary Reporting System (SRS), consistent with the FBI’s annual publication estimates. The variable for property crime includes burglary, larceny, and motor vehicle theft. These types of theft involve unlawfully taking money or property without using force or threatening victims.

Moreover, data on state-level child victimization rates from 2001 to 2019 were sourced from the Child Maltreatment reports published by the Children’s Bureau (Children’s Bureau, 2020). Participation rates for food pantries were derived from a specific question in the Current Population Survey (CPS): ‘Have you received emergency food from a church, food pantry, or food bank in the past month?’ This data is available as part of the Integrated Public Use Microdata Series (IPUMS) (Flood et al., 2022).

To address variation in the percentage of the population who misused OxyContin prior to the reformulation, the treatment variable was measured using data from the National Survey on Drug Use and Health (NSDUH). These data are collected every 2 years, beginning in 2004. The study uses the percentage of nonmedical OxyContin use rates in 2004, 2006, and 2008 as pre-reformulation OxyContin misuse rates. The average of the 2004–2008 nonmedical OxyContin misuse rates was used as the primary fixed misuse rate (National Survey on Drug, 2013).

In addition to NSDUH measures, this study uses an alternative dataset on state-level oxycodone and hydrocodone distribution. Oxycodone is a schedule II narcotic analgesic and can relieve pain for up to 5 h (Drug Enforcement Administration, 2022). Oxycodone and OxyContin are chemically similar, but OxyContin has a longer duration of action. Hydrocodone is another schedule II prescription opioid and the main substitute for oxycodone. Oxycodone and hydrocodone are the main components of prescription opioids in the United States, accounting for 99% of the world’s hydrocodone supply and 83% of the world’s oxycodone (Manchikanti et al., 2012). Data about the legal supply of oxycodone and hydrocodone were collected from the Drug Enforcement Administration (DEA) Automation of Reports and Consolidated Orders System (ARCOS). ARCOS is an automated system developed by the DEA to monitor selected controlled substances at the state level (Automation of Reports and Consolidated, 2023).

State-year level demographic and socioeconomic characteristics were included as covariates to improve estimation precision. The demographic covariates, including race, educational attainment, and age, were collected from the American Community Survey and the Integrated Public Use Microdata Series (Ruggles et al., 2021). Data on population, population density, poverty rates, and unemployment rates were sourced from the University of Kentucky Center for Poverty Research (UKCPR) National Welfare Data.

A potential challenge with the model is that concurrent state policies, such as PDMPs, MMLs, pill mill regulations, and active legal medical marijuana dispensaries, may influence outcomes during the OxyContin reformulation. To address this concern and enhance the precision of the estimates, the analysis controls for these four policy variables, sourced through literature and website searches.

Descriptive statistics for all variables are reported in Table 1. Data are adjusted using state population weights. Subgroup models, such as age-specific arrest rates, use the relevant age-specific state population. Standard errors are clustered at the state level. Data with incomplete information were excluded from the analysis. Prior to the reformulation there were 477.19 arrests for drug abuse violations and 454.36 for drug offenses per 100,000 population. Table 1 also reports the means of demographics characteristics and state-specific economic controls.

**TABLE 1:** Summary statistics of outcomes and key control variables, 2001-2009.

Variable	Mean of all states	Data source
Oxycontin misuse rate (%)	0.567	NSDUH
Oxycodone (doses *per capita*)	0.12	ARCOS
Outcomes (%)		
Arrest rate for drug abuse violations per 100,000	477.19	Crime data explorer
Arrest rate for drug offenses per 100,000	454.36	Crime data explorer
Rates for property crimes per 100,000	3413.21	Crime data explorer, SRS
Rates for burglary per 100,000	734.17	Crime data explorer, SRS
Rates for larceny per 100,000	2293.41	Crime data explorer, SRS
Rates for motor vehicle theft per 100,000	385.62	Crime data explorer, SRS
Child victimization Rate per 1,000 children	11.65	Children’s bureau
Food pantry participation rate	3.25	IPUMS CPS
Demographics characteristics		
Population	5,798,476	UKCPR
Population per square mile	224.19	UKCPR & U.S. Census bureau
Age (%)		
0-19	27.76	U.S. Census bureau
20-39	27.57	U.S. Census bureau
40-64	32.16	U.S. Census bureau
65+	12.51	U.S. Census bureau
Race/ethnicity (%)		
Hispanic	14.59	U.S. Census bureau
Non-Hispanic black	12.24	U.S. Census bureau
Education (%)		
Less than high school education	32.75	IPUMS
High school	22.22	IPUMS
State-specific economic controls (%)		
Poverty rate	12.68	UKCPR
Unemployment rate	5.74	UKCPR
Other policies (%)		
Prescription drug monitoring program (PDMP)	54.55	Prescription drug monitoring Program Training and Technical assistance center; prescription drug abuse policy system; buchmueller and carey (2018); Alpert, Powell, and Pacula (2018)
Pill mill legislation	0.66	Alpert, Powell, and Pacula (2018); Rutkow, Vernick, and Alexander (2017)
Medical marijuana law (MML)	19.81	Alpert, Powell, and Pacula (2018); IIHS
Active and legal medical marijuana dispensaries	8.24	Alpert, Powell, and Pacula (2018); webpage searches
Number of states	51	​

All means are weighted by state population and pooled for 2001–2009 unless otherwise specified. University of Kentucky Center for Poverty Research National Welfare Data (UKCPR). Integrated Public Use Microdata Series (IPUMS). National Survey on Drug Use and Health (NSDUH). Insurance Institute for Highway Safety (IIHS). Summary Reporting System (SRS). Automation of Reports and Consolidated Orders System (ARCOS). Current Population Survey (CPS).

## 3 Identification strategy

Studying a nationwide policy introduced simultaneously across all states is challenging because there is no cross-sectional variation in the timing of policy implementation (Beheshti, 2019). OxyContin misuse prior to the reformulation does, however, differ across states. We use this variation to examine whether the OxyContin reformulation increased drug-related arrest rates and property-related crime rates. The underlying assumption is that states with low OxyContin misuse rates prior to the reformulation were less affected, while states with high misuse rates were more significantly impacted. Alpert et al. (2018) developed this methodology, which exploits variation in the prevalence of OxyContin misuse at the state level prior to the policy’s introduction (Alpert et al., 2018). Building on Alpert’s study, this paper incorporates new perspectives on crime and arrest rates, as well as more recent data, to demonstrate longer-term effects. Recent drug-related violations are increasingly concentrated in suburban and rural areas compared to past drug problems. Furthermore, nearly 18% of child fatalities and 25% of child abuse victims were associated with a caregiver who abused drugs based on the child maltreatment report 2015 (Child Maltreatment, 2017). And the percentage of victims increased to 29% in 2019 (Child Maltreatment, 2021). This study will fill in the gaps left by Alpert.

**FIGURE 2:**
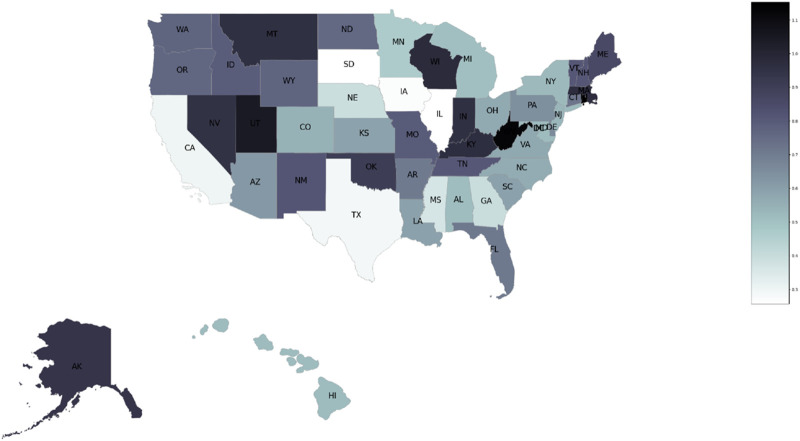
State variation in OxyContin misuse rates prior to the reformulation. Notes: Pre-reformulation OxyContin misuse rates measured by the average of 2004, 2006, and 2008 nonmedical OxyContin misuse rates in the National Survey on Drug Use and Health (NSDUH).

This identification was first introduced by Card (Card, 1992), who used the percentage of workers earning less than the minimum wage in each state before the minimum wage policy was implemented as the policy’s treatment measure (Gladden et al., 2016; Rudd et al., 2014). Figure 2 depicts OxyContin misuse rates prior to the reformulation in all states. Darker shaded states reported higher OxyContin misuse rates prior to the reformulation, and lighter shaded states reported lower OxyContin misuse rates. State misuse rates were calculated as the average of nonmedical OxyContin use rates in 2004, 2006, and 2008. Using average values reduces measurement error associated with a single wave. In addition, using data that are not close to the time of treatment reduces concerns about measurement error in misuse rates, since mixed situations may occur close to the treated year. For instance, some abusers might stock up on the original formulation in advance if they know the drug will be reformulated.

Next, we use difference-in-differences (DID) to estimate the causal effects of the OxyContin reformulation on arrest rates for drug abuse violations and drug offenses and rates for property crimes, burglary, larceny, and motor vehicle theft. The DID framework can isolate the causal relationship between the reformulation, drug-related-arrest rates and property-related-crimes rates. In addition, the event study specifications can provide evidence of whether the arrest rates trend together prior to the treatment and whether a trend break occurs after the reformulation in 2010. The model can be specified as follows.

$$Y_{st}=\sum_{t\neq 2009}\beta_{t}{OxyRate}_{s}^{pre}+{\delta X}_{st}+\alpha_{s}+\gamma_{t}+\varepsilon_{st}$$ (1)

where $Y_{st}$ indicates the arrest rates for drug abuse violations and drug offenses and rates for property crimes, burglary, larceny, and motor vehicle theft in state s in year t; $\beta_{t}{OxyRate}_{s}^{pre}$ is the interaction of the fixed rate of OxyContin misuse in state s prior to the reformulation was implemented and a full set of year fixed effects. $X_{st}$ is a vector for state and time-varying covariates: log population, population per square mile, share non-Hispanic black, share Hispanic, share of the population with at least a high school degree, share of the population with less than a high school degree, population density, share of multiple age groups (0-19, 20-39, 65+), poverty rate, and unemployment rate. State policy variables accounted for include indicators for prescription drug monitoring programs (PDMPs), pill mill legislation, medical marijuana laws (MMLs), and active legal medical marijuana dispensaries. $\alpha_{s}$ controls for state fixed effects; $\gamma_{t}$ controls for year fixed effects; the reference group is 2009, 1 year prior to the reformulation was introduced; and $\beta_{t}$ is the differences between the trends in other years and the trend in 2009. If there are no differences in the trend of arrest rates prior to the reformulation and trend breaks after the reformulation, $\beta_{t}$ should be close to 0 prior to the reformulation was launched in 2010 and increase after 2010.

**FIGURE 3:**
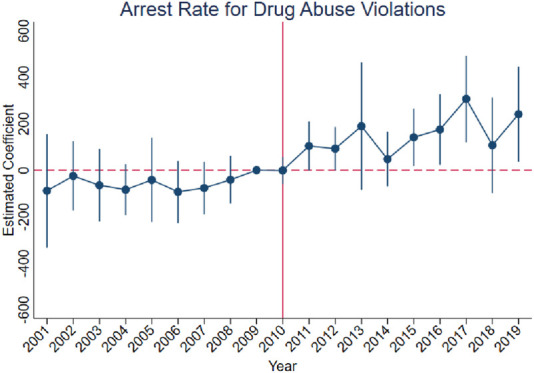
Effect of OxyContin reformulation on arrest rates for drug abuse violations per 100,000 population—event study specification. No*tes:* Figures display point estimates and corresponding 95% confidence intervals. 2009 is the reference year.

**FIGURE 4:**
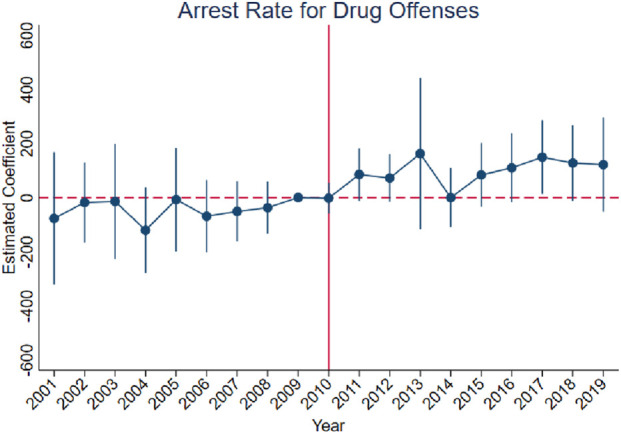
Effect of OxyContin reformulation on arrest rates for drug offenses per 100,000 population—event study specification. Notes: Figures display point estimates and corresponding 95% confidence intervals. 2009 is the reference year.

**FIGURE 5:**
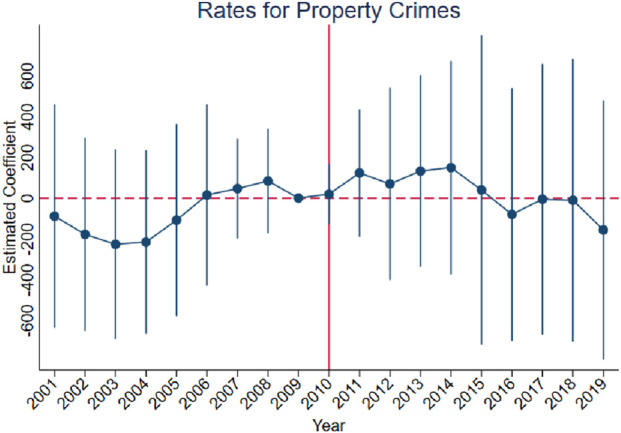
Effect of OxyContin reformulation on rates of property crimes per 100,000 population—event study specification. Notes: Figures display point estimates and corresponding 95% confidence intervals. 2009 is the reference year.

**FIGURE 6:**
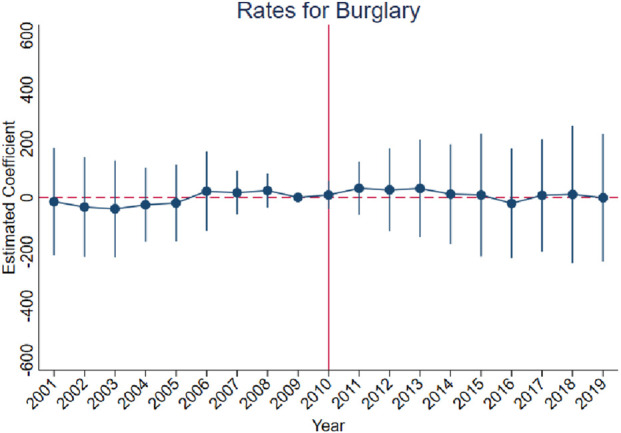
Effect of OxyContin reformulation on rates of burglary per 100,000 population—event study specification. Notes: Figures display point estimates and corresponding 95% confidence intervals. 2009 is the reference year.

**FIGURE 7:**
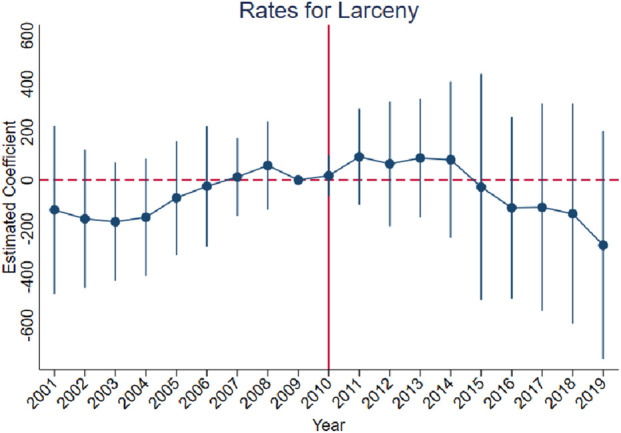
Effect of OxyContin reformulation on rates of larceny-thefts per 100,000 population—event study specification. Notes: Figures display point estimates and corresponding 95% confidence intervals. 2009 is the reference year.

**FIGURE 8:**
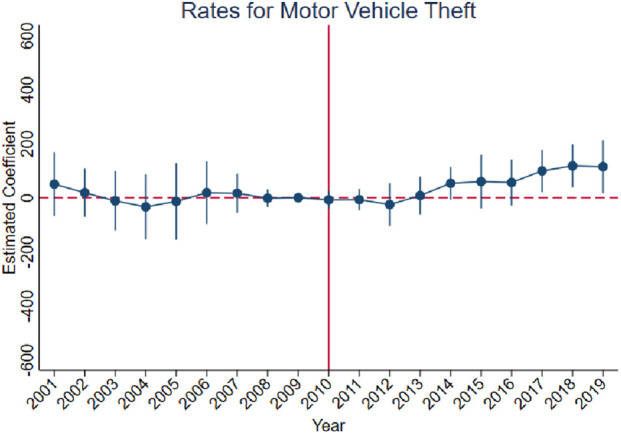
Effect of OxyContin reformulation on rates of motor vehicle theft per 100,000 population—event study specification. Notes: Figures display point estimates and corresponding 95% confidence intervals. 2009 is the reference year.

**FIGURE 9:**
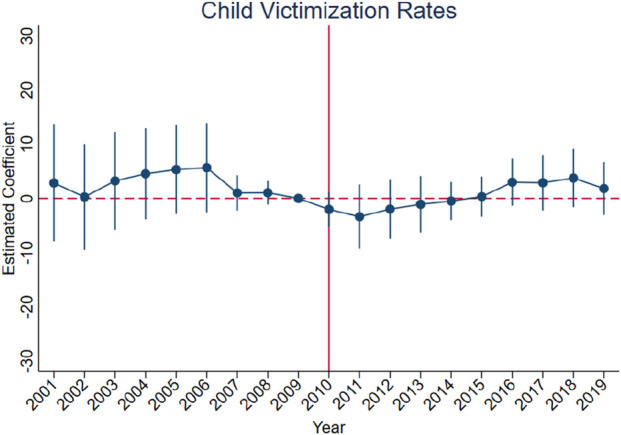
Effect of OxyContin reformulation on child victimization rate per 1,000 Children—event study specification. Notes: Figures display point estimates and corresponding 95% confidence intervals. 2009 is the reference year.

**FIGURE 10:**
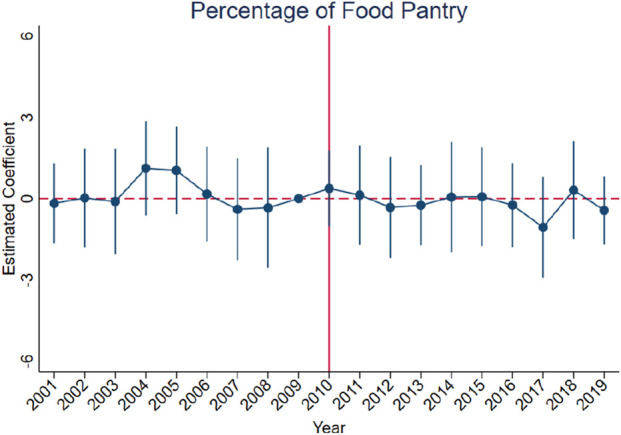
Effect of OxyContin reformulation on food pantry participation rate—event study specification. Notes: Figures display point estimates and corresponding 95% confidence intervals. 2009 is the reference year.

Figures 3–10 show that, compared to states with low OxyContin misuse rates, trends in states with high misuse rates are statistically insignificant in the years preceding the reformulation. Specifically, a regression that interacts pre-reformulation OxyContin misuse rates with the set of year dummy variables shows no significant differences for all coefficients prior to the reformulation compared with the reference year of 2009. Thus, substantial evidence from the event studies specifications supports a casual interpretation of results from the DID model.

Following Alpert et al. methodology (Alpert et al., 2018), we restrict the basic DID model and use a trend-break DID framework by including a level shift, a preexisting trend, and a trend break in the model.

$$Y_{st}=\beta_{1}\left[{OxyRate}_{s}^{pre}\;*\;{Post}_{t}\right]+\beta_{2}\left[t\;*\;\;{OxyRate}_{s}^{pre}\right]+\beta_{3}\left[{{Post}_{t}\;*\;\left(t-2011\right)\;*\;OxyRate}_{s}^{pre}\right]+{\delta X}_{st}+\alpha_{s}+\gamma_{t}+\varepsilon_{st}$$ (2)

where $\beta_{1}\left[{OxyRate}_{s}^{pre}\;*\;{Post}_{t}\right]$ controls for a level shift; $\beta_{2}\left[t\;*\;\;{OxyRate}_{s}^{pre}\right]$ controls for a preexisting trend; and $\beta_{3}\left[{{Post}_{t}\;*\;\left(t-2011\right)\;*\;OxyRate}_{s}^{pre}\right]$ controls for a trend break. Because the OxyContin reformulation was introduced in late 2010 and pharmacies might have inventories that include the original formulation, we set 2011 as the first year the reformulation was introduced. The primary benefit of this model is that it enables me to examine the effects in various time spans. $\beta_{1}$ is the effect of the reformulation through 2011; $\beta_{1}+\beta_{3}$ is the effect through 2012; and $\beta_{1}+{2\beta }_{3}$ is the effect through 2013. Based on data availability, we can obtain the average effect of the reformulation until 2019.

### 3.1 Potential threat

A potential threat to identifying the causal relationship using variation in OxyContin misuse rates prior to the reformulation is the lack of randomization across states, a fundamental limitation of the DID method. For instance, high OxyContin misuse may be associated with more illicit market trade and less expenditure on police, which might lead more abusers to commit crimes. Although we control for state policy variables such as PDMPs, pill mill legislation, MMLs, and active legal medical marijuana dispensaries, other drug- or crime-related policies implemented concurrently could weaken the causal link between the reformulation and crime outcomes.

To overcome those potential threats and construct a more convincing causal relationship, in addition to exploiting the event study specifications, we will create three tertiles/groups based on the OxyContin misuse rates: states with low OxyContin misuse rates, states with medium OxyContin misuse rates, and states with high OxyContin misuse rates. According to Rosenbaum’s (2010) argument including control or comparison groups could meaningfully strengthen the effects caused by the treatment (Rosenbaum and Rosenbaum, 2010). The hypothesis is that states with high OxyContin misuse rates will have higher crime rates than those with low misuse rates, while states with medium misuse rates will fall between the two. Combined with the assumption about the OxyContin reformulation, we hope to see that states with high OxyContin misuse rates are more affected by the reformulation than states with low misuse rates, and moderate effects on states with medium misuse rates. The model can be specified as follows.

$$Y_{st}=\beta_{1}OxyC_{s}\;*\;Post_{t}+{\delta X}_{st}+\alpha_{s}+\gamma_{t}+\varepsilon_{st}$$ (3)

where *OxyC*_*s*_ is a dichotomous measure on whether state *s* in the treatment group of states with high misuse rates, or in the control group of states with medium misuse rates, or the group of states with low misuse rates prior to the reformulation. The tertiles for OxyContin misuse were constructed using the 33rd and 67th percentiles of the pre-reformulation distribution of misuse rates. *Post*_*t*_ is an indicator that equals to one if the year is 2011 and later. *β*_1_ represents the average effects of OxyContin reformulation.

## 4 Results

The results are presented in four steps, based on the research design described above. First, we assess the validity of the parallel trends assumption using event study specifications (Equation 1), with results presented in Figures 3–10. Second, we present baseline estimates from a standard difference-in-differences model using a dichotomous treatment indicator, as specified in Equation 3. These results, reported in Table 2 and Supplementary Table A1 (Appendix), summarize average post-reformulation differences in outcomes across states grouped by pre-reformulation misuse rate. Third, we estimate our primary effects using the trend-break difference-in-differences framework in Equation 2, following Alpert et al. (2018). The results, reported in Table 3 and Supplementary Table A2 (Appendix), capture the dynamic effects of the OxyContin reformulation over time while accounting for pre-existing trends. Finally, we perform multiple falsification tests as robustness checks, which add additional support for the primary results.

**TABLE 2:** Effect of OxyContin reformulation on the outcomes, dichotomous treatment model.

​	Low OxyContin misuse vs. high OxyContin misuse			Low OxyContin misuse vs. medium OxyContin misuse		
	Baseline model	Baseline model + state and time-varying covariates	Preferred model: baseline model + state and time-varying covariates + policy variables	Baseline model	Baseline model + state and time-varying covariates	Preferred model: baseline model + state and time-varying covariates + policy variables
Arrest rates for drug abuse violations per 100,000	129.00**	97.70***	111.69***	78.03	49.80	60.25*
	(47.98)	(31.44)	(37.44)	(46.41)	(33.10)	(34.31)
R^2^	0.763	0.797	0.796	0.750	0.786	0.786
N	645	645	627	630	630	606
Number of states + DC	34	34	34	34	34	34
State and time-varying covariates	No	Yes	Yes	No	Yes	Yes
Policy variables	No	No	Yes	No	No	Yes
Arrest rates for drug offenses per 100,000	113.06**	86.53***	92.55***	63.03	41.65	49.44
	(50.36)	(28.01)	(30.4)	(48.31)	(31.84)	(32.38)
R^2^	0.824	0.857	0.858	0.844	0.877	0.879
N	645	645	627	630	630	606
Number of states + DC	34	34	34	34	34	34
State and time-varying covariates	No	Yes	Yes	No	Yes	Yes
Policy variables	No	No	Yes	No	No	Yes
Rates for motor vehicle theft per 100,000	53.21*	27.63	17.4	−6.99	5.46	−4.15
	(26.76)	(26.12)	(23.29)	(33.62)	(20.05)	(19.72)
R^2^	0.892	0.926	0.936	0.876	0.917	0.926
N	646	646	628	646	646	622
Number of states + DC	34	34	34	34	34	34
State and time-varying covariates	No	Yes	Yes	No	Yes	Yes
Policy variables	No	No	Yes	No	No	Yes

***p < 0.01, **p < 0.05, *p < 0.1. Figures in parentheses are the respective robust standard errors, clustered at state level. N reports state-year observations. The dichotomous treatment is defined based on the tertiles of OxyContin misuse at the state level prior to the reformulation. States are categorized into 'Low', 'Medium', and 'High' OxyContin misuse groups. State fixed effects and year fixed effects are included in all specifications. State and time-varying covariates include log population, population per square mile, share non-Hispanic Black, share Hispanic, share with high school degree, share with less than high school degree, share with multiple age groups (0-19, 20-39, 65+), poverty rate, and unemployment rate. We also account for state policy variables including indicators for prescription drug monitoring programs, pill mill legislation, medical marijuana laws, and active and legal medical marijuana dispensaries. Regressions are weighted by population. Years 2001–2019 are used. Significance levels are indicated as follows: *p* < 0.01, *p* < 0.05, and p < 0.1.

**TABLE 3:** Effect of OxyContin reformulation on state arrests for drug abuse violation rates and motor vehicle theft, trend-break model.

​	Arrest Rates for Drug Abuse Violations per 100,000				Rates for Motor Vehicle Theft per 100,000			
	Linear time trend interaction	Linear time trend interaction + state and time-varying covariates	State and time-varying covariates + policy variables	Preferred model: linear time trend interaction + state and time-varying covariates + policy variables	Linear time trend interaction	Linear time trend interaction + state and time-varying covariates	State and time-varying covariates + policy variables	Preferred model: linear time trend interaction + state and time-varying covariates + policy variables
1-year effect	106.22	88.35	140.21**	106.40	11.25	0.99	-23.17	-10.82
	(84.38)	(70.11)	(64.75)	(69.47)	(18.55)	(27.26)	(32.55)	(30.58)
2-year effect	100.01	95.89	156.15**	115.68*	21.03	18.92	-4.70	10.03
	(77.38)	(67.43)	(59.43)	(68.89)	(16.55)	(27.74)	(29.13)	(30.28)
3-year effect	99.80	103.44	172.08***	124.96*	30.80*	38.84	13.76	30.88
	(72.51)	(68.47)	(56.42)	(71.68)	(17.02)	(30.38)	(27.21)	(32.81)
4-year effect	87.59	110.99	188.02***	134.24*	40.58**	58.76*	32.22	51.73
	(70.18)	(73.08)	(56.11)	(77.47)	(19.79)	(34.71)	(27.11)	(37.58)
5-year effect	81.38	118.53	203.95***	143.51*	50.35**	78.67*	50.68*	72.58
	(70.66)	(80.64)	(58.54)	(85.66)	(24.09)	(40.17)	(28.85)	(43.88)
6-year effect	75.18	126.08	219.88***	**152.79**	60.13**	98.59**	69.15**	93.43*
	(73.90)	(90.42)	(63.39)	(95.64)	(29.24)	(46.36)	(32.14)	(51.14)
7-year effect	68.97	133.62	235.82***	**162.07**	69.90**	118.51**	87.61**	114.28*
	(79.55)	(101.79)	(70.16)	(106.90)	(34.87)	(53.04)	(36.56)	(59.00)
8-year effect	62.76	141.17	251.75***	**171.35**	79.68*	138.42**	106.07**	135.13**
	(87.16)	(114.26)	(78.36)	(119.08)	(40.78)	(60.04)	(41.75)	(67.27)
9-year effect	56.55	148.71	267.69***	**180.63**	89.45*	158.34**	124.54**	155.98**
	(96.25)	(127.51)	(87.58)	(131.93)	(46.87)	(67.26)	(47.46)	(75.80)
R2	0.735	0.768	0.764	0.764	0.863	0.905	0.911	0.911
N	952	952	922	922	969	969	939	939
Number of states + DC	51	51	51	51	51	51	51	51
State and time-varying covariates	No	Yes	Yes	Yes	No	Yes	Yes	Yes
Linear time trend interaction	Yes	Yes	No	Yes	Yes	Yes	No	Yes
Policy variables	No	No	Yes	Yes	No	No	Yes	Yes

*** p<0.01, ** p<0.05, * p<0.1. Figures in parentheses are the respective robust standard errors, clustered at state level. N reports state-year observations. Except for models (3), (7), (11), (15), (19) and (23), each model also includes a post indicator interacted with initial OxyContin misuse, a linear time trend interacted with initial OxyContin misuse, and a post-2011 linear trend interacted with initial OxyContin misuse. State fixed effects and year fixed effects are included in all specifications. State and time-varying covariates include log population, population per square mile, share non-Hispanic Black, share Hispanic, share with high school degree, share with less than high school degree, share with multiple age groups (0-19, 20-39, 65+), poverty rate, and unemployment rate. We also account for state policy variables including indicators for prescription drug monitoring programs, pill mill legislation, medical marijuana laws, and active and legal medical marijuana dispensaries. Regressions are weighted by population. Years 2001–2019 are used. Significance levels are indicated as follows: *p* < 0.01, *p* < 0.05, and p < 0.1.

We begin by using the event study framework in Equation 1 to test the underlying DID assumption. Figures 3–10 show that trends for states with high OxyContin misuse rates are statistically insignificant in the years prior to introduction of the OxyContin reformulation, compared with states that have with low OxyContin misuse rates. Specifically, a regression that interacts pre-reformulation OxyContin misuse rates with the set of year dummy variables shows that there were no significant differences for all coefficients prior to the reformulation compared with the reference year of 2009. Joint F-tests confirm that pre-reformulation coefficients are jointly insignificant. Thus, strong evidence from the event studies specifications supports a casual interpretation of results from the DID model. Furthermore, the findings from the dichotomous treatment model in Table 2 and Supplementary Table A1 confirm the hypothesis that states with high OxyContin misuse rates are more affected by the reformulation than states with low misuse rates, and moderate effects on states with medium misuse rates.

Table 2 presents the baseline effects of OxyContin reformulation on outcomes throughout the entire post-reformulation period. The results indicate that drug reformulation significantly increased in the arrest rates for drug abuse violations (by 111.69 percentage points when comparing states with low to high OxyContin misuse rates, and a 60.25 percentage point increase when comparing states with low to medium misuse rates). These changes occurred throughout the entire post-reformulation period. The results were derived from models that included controls for various state demographic characteristics and policy variables that underwent modifications over time - such as Prescription Drug Monitoring Programs (PDMP), pill mill legislation, Medical Marijuana Laws (MML), and active and legal medical marijuana dispensaries. The findings substantiate the hypothesis that the reformulation of OxyContin had a more pronounced impact on states with high misuse rates, while exerting a moderate effect on states with medium misuse rates.

Table 3 presents our primary results from the trend-break DID model. The first four columns report the effects of the OxyContin reformulation on arrest rates for drug abuse violations. We find that the reformulation is associated with higher arrest rates from years one through 9 (column 3), after controlling for state- and time-varying covariates and policy variables.

Effect sizes are interpreted per one-percentage-point increase in pre-reformulation OxyContin misuse. Estimates from column 3 indicate that a one-percentage-point increase in the rate of OxyContin misuse prior to the reformulation increases arrests for drug abuse violations per 100,000 population, rising from 156.15 in 2012 to 203.95 in 2015. After adding yearly trends interacted with fixed rates of OxyContin misuse in states to control for preexisting trends, we find positive effects for years two through 5 (column 4). Estimates in the model that controls for demographic characteristics, other policies, and preexisting trends indicate that a one-percentage-point increase in the rate of OxyContin misuse prior to the reformulation increases arrests for drug abuse violations per 100,000 population, rising from 115.68 in 2012 to 143.51 in 2015. The coefficients are relatively large compared to the mean arrest rates for drug abuse violations prior to the reformulation. The results show that arrest rates for drug abuse violations per 100,000 increased by about 24% in 2012, 26% in 2013, 28% in 2014, and 30% in 2015.

As with the point estimates in columns 5 to 8 of Table 3, the point estimates in these columns suggest that the reformulation differentially increased motor vehicle theft with higher initial OxyContin misuse; positive impacts of the OxyContin reformulation on motor vehicle theft were found with different models. Even if we estimate using the most restricted model (column 8), these four significant results were still found. In Column 8, a state with 1 percentage point more OxyContin misuse in the pre-period would expect 93.43 more motor vehicle thefts per 100,000 population in 2016; 114.28 in 2017; 135.13 in 2018; and 155.98 in 2019.

Results for arrest rates for drug offenses are shown in Supplementary Table A2. We find significant effects on arrest rates for drug offenses in the model that controls for demographic characteristics and other policies but not for preexisting trends. However, we find no statistically significant effects on arrest rates for drug offenses after controlling for preexisting trends.

Supplementary Table A2 also present the results for rates of property crimes, burglary, and larceny. Without preexisting trends, we observe significant impacts of the OxyContin reformulation on rates for property crimes and larceny. These effects are no longer statistically significant after accounting for preexisting trends.

In addition, we find no statistically significant effects of the reformulation on child victimization rates or food pantry participation (Supplementary Table A2). These findings are consistent across various models and analytical approaches, possibly due to the nonspecific nature of the variables and the indirect effects of the reformulation. It is also possible that these outcomes respond with a longer lag than arrests or property crimes, as child maltreatment and food insecurity often take more time to be recorded. A more suitable variable, such as 'child victims with a drug-abusing caregiver' or ‘food pantry participation with drug-abusing family member’, could perhaps provide more targeted insights. However, relevant data from the children’s bureau and CPS is not available for the analysis period. Furthermore, long-term OxyContin abusers may not be significantly affected by the reformulation, suggesting that these findings do not necessarily indicate a worsening situation, but rather a stable status regarding child victimization rates and food pantry participation rates.

We also find Important differences in the effects of the reformulation across subgroups by age emerged for drug abuse violation arrest rates. Table 4 presents estimate for four age subgroups. The results show that the OxyContin reformulation significantly increased arrest rates for drug abuse violations among individuals aged 40–64. No statistically significant effects of the reformulation were observed in other age groups. For example, a state with 1 percentage point more OxyContin misuse in the pre-period would expect 83.83 and 98.89 more drug abuse violations per 100,000 population in 2012 and 2013 for individuals ages 40-64.

**TABLE 4:** Heterogeneity in effects of arrest rates for drug abuse violations.

Outcome	Arrest rates for drug abuse violations per 100,000			
By subgroup	Age group			
	Ages 0-19	Ages 20-39	Ages 40-64	Ages 65+
1-year effect	21.95	219.80	68.77	4.28
	(84.13)	(159.08)	(48.64)	(4.04)
2-year effect	32.29	206.42	83.83*	3.85
	(89.99)	(156.27)	(48.95)	(3.70)
3-year effect	42.62	193.04	98.89*	3.41
	(96.37)	(164.05)	(51.45)	(3.43)
4-year effect	52.95	179.65	113.95**	2.98
	(103.16)	(181.06)	(55.84)	(3.22)
5-year effect	63.28	166.27	129.01**	2.54
	(110.28)	(205.02)	(61.73)	(3.11)
6-year effect	73.61	152.89	144.07**	2.11
	(117.68)	(233.81)	(68.73)	(3.10)
7-year effect	83.95	139.51	159.12**	1.67
	(125.31)	(265.85)	(76.53)	(3.19)
8-year effect	94.28	126.13	174.18**	1.23
	(133.13)	(300.11)	(84.91)	(3.38)
9-year effect	104.61	112.75	189.24**	0.80
	(141.11)	(335.91)	(93.73)	(3.64)
Mean of outcome	326.76	961.86	258.06	9.91

***p < 0.01, **p < 0.05, *p < 0.1. Figures in parentheses are the respective robust standard errors, clustered at state level. Each model includes a post indicator interacted with initial OxyContin misuse, a linear time trend interacted with initial OxyContin misuse, and a post-2011, linear trend interacted with initial OxyContin misuse. State fixed effects and year fixed effects are included in all specifications. State and time-varying covariates include log population, population per square mile, share non-Hispanic Black, share Hispanic, share with high school degree, share with less than high school degree, share with multiple age groups (0-19, 20-39, 65+), poverty rate, and unemployment rate. We also account for state policy variables including indicators for prescription drug monitoring programs, pill mill legislation, medical marijuana laws, and active and legal medical marijuana dispensaries. Regressions are weighted by age-specific population. Years 2001–2019 are used.

## 5 Robustness tests

To determine whether our findings are robust, we conduct multiple falsification tests and a robustness test using alternative ARCOS data. First, we use pre-reformulation rates of pain reliever misuse instead of OxyContin misuse rates to test the assumption that only the latter reflects state-level variations in the reformulation. Pre-reformulation nonmedical pain reliever rates, being more general, are not expected to reflect state-level variations in the reformulation or affect arrest rates for drug abuse violations and motor vehicle theft rates. Second, we estimate the effects of the OxyContin reformulation on all violent crimes. The variable for all violent crime is composed of four offenses: homicide, rape, robbery, and aggravated assault. Heroin, which is a depressant, is commonly considered to be negatively related to violent crimes. In addition, the variable for all violent crimes is a general variable similar to the nonmedical pain reliever rate. Thus, we hypothesize that there is negative or no significant effects on all violent crimes in our analysis of the OxyContin reformulation. Third, we implement a temporal falsification test where we estimate the model using a false temporal policy cutoff year in 2006. The results of the temporal falsification test are shown in Table 5.

**TABLE 5:** Falsification tests - Effect of OxyContin reformulation on state arrests for drug abuse violation rates and motor vehicle theft using a false temporal policy cutoff in 2006, trend-break model.

Outcomes	Arrest rates for drug abuse violations per 100,000	Rates for motor vehicle theft per 100,000
False year: 2006		
1-year effect	5.59	60.47
	(52.64)	(50.98)
2-year effect	−0.45	81.83
	(64.29)	(75.01)
3-year effect	−6.49	103.19
	(80.33)	(101.32)
4-year effect	−12.52	124.56
	(98.65)	(128.52)
5-year effect	−18.56	145.92
	(118.18)	(156.14)
R^2^	0.880	0.941
N	499	510
Number of states + DC	51	51
State and time-varying covariates	Yes	Yes
Linear time trend interaction	Yes	Yes
Policy variables	Yes	Yes

***p < 0.01, **p < 0.05, *p < 0.1. Figures in parentheses are the respective robust standard errors, clustered at state level. Each model includes a post indicator interacted with initial OxyContin misuse, a linear time trend interacted with initial OxyContin misuse, and a post-2011, linear trend interacted with initial OxyContin misuse. State fixed effects and year fixed effects are included in all specifications. State and time-varying covariates include log population, population per square mile, share non-Hispanic Black, share Hispanic, share with high school degree, share with less than high school degree, share with multiple age groups (0-19, 20-39, 65+), poverty rate, and unemployment rate. We also account for state policy variables including indicators for prescription drug monitoring programs, pill mill legislation, medical marijuana laws, and active and legal medical marijuana dispensaries. Regressions are weighted by population. Years 2001–2010 are used. The false temporal policy cutoff year is 2006. Significance levels are indicated as follows: *p* < 0.01, *p* < 0.05, and p < 0.1.

**FIGURE 11:**
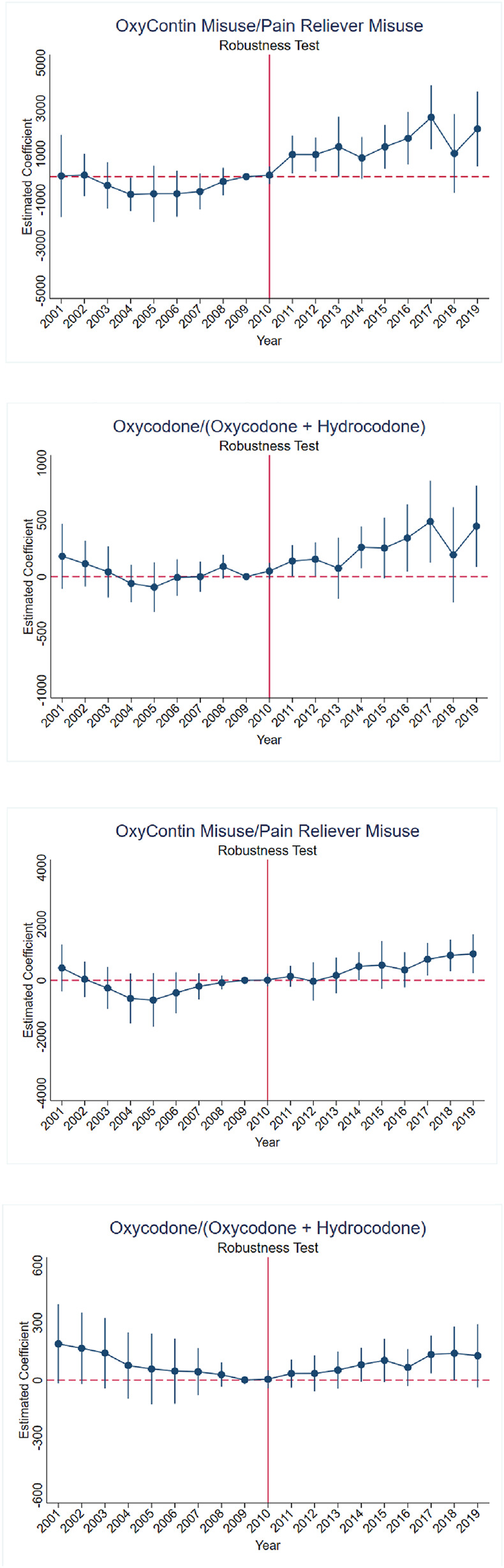
Robustness test—event study results for arrest rates for drug abuse violations and rates for motor vehicle theft using alternative measures of exposure to the reformulation **(A)** Arrest rates for drug abuse violations using OxyContin misuse/pain reliever misuse. **(B)** Arrest rates for drug abuse violations using Oxycodone/(oxycodone + hydrocodone). **(C)** Rates for motor vehicle theft using OxyContin misuse/pain reliever misuse. **(D)** Rates for motor vehicle theft using Oxycodone/(oxycodone + hydrocodone). *Notes*: Each graph includes point estimates from event study (normalized to 0 in 2009) and 95 percent confidence intervals that are adjusted for within-state clustering. Panel A and C uses NSDUH data, panels B and D use ARCOS data.

Finally, we use an alternative dataset on oxycodone and hydrocodone variations to validate the accuracy of OxyContin misuse measures from the NSDUH. We present the results in Figure 11 and Table 6. Since the identifying assumption of the study relies on OxyContin misuse rates, directly replacing them with the legal supply of oxycodone is not appropriate. Thus, we established two additional measures of exposure to the reformulation, one as the fraction of the initial rate of OxyContin misuse divided by the initial rate of pain reliever, and the another as the fraction of the initial supply of oxycodone divided by the total supply of oxycodone and hydrocodone combined, and then compared them with each other and with the main results. Since the proportion of OxyContin among all pain relievers is much lower than the proportion of oxycodone in only oxycodone and hydrocodone combined, in other words, the units are different for these exposure measures, we exploit the effect of a one standard deviation increase in exposure to the reformulation as the standard magnitudes of estimates.

**TABLE 6:** Robustness test - alternative measures of exposure to the reformulation.

Panel A	Arrest rates for drug abuse violations per 100,000		
	Main results	OxyContin Misuse/Pain reliever misuse	Oxycodone/(oxycodone + hydrocodone)
2-year effect	115.68*	1246.95**	169.24**
	(68.89)	(499.99)	(82.60)
3-year effect	124.96*	1373.95**	214.30**
	(71.68)	(522.06)	(92.20)
4-year effect	134.24*	1500.95***	259.37**
	(77.47)	(562.97)	(105.55)
5-year effect	143.51*	1627.96**	304.43**
	(85.66)	(618.99)	(121.42)
Effect of one Std Dev increase	32.01	50.17	50.73

Panel B	Rates for motor vehicle theft per 100,000		
	Main results	OxyContin Misuse/Pain reliever misuse	Oxycodone/(oxycodone + hydrocodone)
6-year effect	93.43*	829.39**	243.67***
	(51.14)	(360.68)	(88.27)
7-year effect	114.28*	953.54**	278.55***
	(59.00)	(405.62)	(88.27)
8-year effect	135.13**	1077.68**	313.43***
	(67.27)	(456.11)	(99.35)
9-year effect	155.98**	1201.83**	348.31***
	(75.80)	(510.52)	(110.90)
Effect of one Std Dev increase	34.79	37.03	58.04

***p < 0.01, **p < 0.05, *p < 0.1. Figures in parentheses are the respective robust standard errors, clustered at state level. Each model includes a post indicator interacted with initial OxyContin misuse, a linear time trend interacted with initial OxyContin misuse, and a post-2011, linear trend interacted with initial OxyContin misuse. State fixed effects and year fixed effects are included in all specifications. State and time-varying covariates include log population, population per square mile, share non-Hispanic Black, share Hispanic, share with high school degree, share with less than high school degree, share with multiple age groups (0-19, 20-39, 65+), poverty rate, and unemployment rate. We also account for state policy variables including indicators for prescription drug monitoring programs, pill mill legislation, medical marijuana laws, and active and legal medical marijuana dispensaries. Regressions are weighted by population. Years 2001–2019 are used. Since units are different for each exposure measure, the effect of a one standard deviation increase in exposure to the reformulation is shown in the bottom row. Significance levels are indicated as follows: *p* < 0.01, *p* < 0.05, and p < 0.1.

**FIGURE 12:**
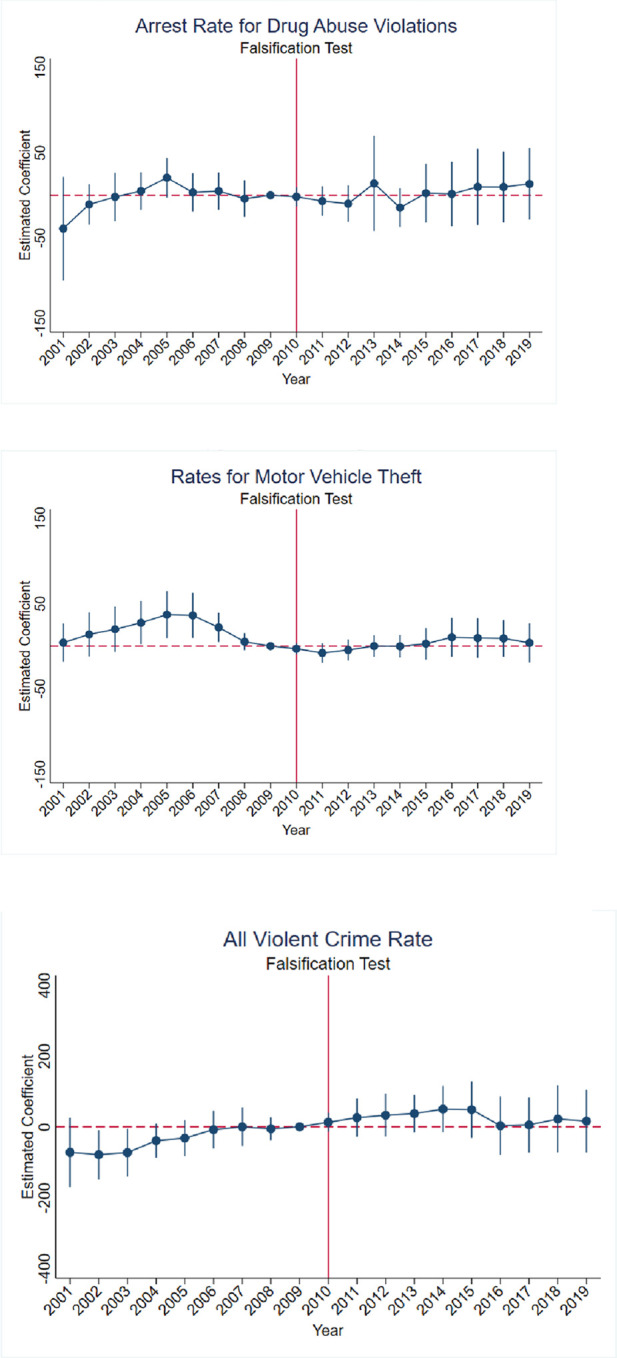
Falsification tests—effects of OxyContin reformulation on arrest rates for drug abuse violations and all violent crime rates per 100,000 population—event study specification **(A)** Arrest rates for drug abuse violations using pre-reformulation pain reliever misuse rates. **(B)** Rates for motor vehicle theft using pre-reformulation pain reliever misuse rates. **(C)** All violent crimes rates using pre-reformulation OxyContin misuse rates. Notes: Figures display point estimates and corresponding 95% confidence intervals. 2009 is the reference year.

Figure 12 shows the event study specifications for the robustness of our findings. Panels A and B of Figure 12 present estimates using pre-reformulation pain reliever misuse rates, while Panel C shows estimates for all violent crime rates. As we expected, estimates from the falsification tests are not significant for Panels A, B and C. These results provide evidence that individuals misusing other prescription pain relievers such as Vicodin, which were not affected by the OxyContin reformulation, were also impacted. In addition, the OxyContin reformulation should only have effects on drug abuse violations and other crimes related to money associated with abusers who transferred from less costly OxyContin to the higher cost of illegal drugs.

Table 5 shows that no significant results were found when we artificially move the date of the OxyContin reformulation back in time from 2010 to 2006. The data, consistent back to 2001, allows for a full 5-year pre- and post-reformulation time window. he results suggest that effects on the arrest rates for drug abuse violations and the rates for motor vehicle theft did not exist prior to the actual implementation of reformulation in 2010.

In Panels A and C of Figure 11, the treatment variable is defined as the fraction of initial OxyContin misuse divided by initial pain reliever misuse. And we exploit the fraction of the initial supply of oxycodone divided by the total supply of oxycodone and hydrocodone combined as the measure of treatment variables in Panel B and D of Figure 11. The pattern of event studies is very similar between panels A and B in Figure 11, as well as between panels C and D in Figure 11. In addition, the magnitudes of estimates for a one standard deviation increase showing in Table 6 are stable across measures as well. The robustness test indicates that results using the NSDUH OxyContin misuse measures are reliable and insensitive to different datasets.

## 6 Discussion

This study examines the unintended impacts of the OxyContin reformulation on drug-related arrest rates and property crimes. Using a causal inference design, including a DID method and event study specifications, we find that the OxyContin reformulation increased arrest rates for drug abuse violations in both the short and long term. A long-term state-year-level dataset (2001–2019) enables investigation of the long-term impacts of the reformulation.

### 6.1 Arrest rates for drug abuse violations

In this paper, we provide evidence on the relationship between the OxyContin reformulation and drug abuse violations. Our analysis employs a trend-break DID identification strategy, enabling a causal assessment of the reformulation’s impact. Multiple falsification tests support the robustness of the results. A key strength of this study lies in its comprehensive approach, leveraging state-year-level data constructed from diverse sources to provide detailed insights.

Most previous studies’ analyses are restricted to estimates of short-term effects (Alpert et al., 2018; Evans et al., 2019). In contrast, this study examines the effects in both the short and long term; we use a long-range state-year-level dataset and rich variables to improve the overall precision of estimates.

### 6.2 Motor vehicle theft

While no significant direct impacts were found on property crimes, our analysis highlights motor vehicle theft, which constitutes a significant portion of property crimes. we find that motor vehicle theft increased after the OxyContin reformulation, which is consistent with previous study findings that heroin use is positively associated with property crimes (Anglin and Speckart, 1988; Barton, 1980). This finding also suggests that the OxyContin reformulation may have led to increased heroin use, which in turn contributed to higher motor vehicle theft rates. Furthermore, the long-term effects of the reformulation on motor vehicle theft (2016–2019) are consistent with observed behavioral patterns, whereby individuals with opioid use disorder may drain their savings in certain years and subsequently engage in property crimes.

### 6.3 Age-specific effects

The specific finding that drug abuse violations due to the reformulation increase only for individuals ages 40-64 is consistent with Case and Deaton’s findings (Case and Deaton, 2015); the 40-64 age group accounts for the largest portion of the labor force and is also the group most affected by the opioid epidemic.

## 7 Limitations and perspectives

The study is subject to several limitations. One limitation is that OxyContin misuse rates prior to the reformulation were collected at the state and year levels, but estimates might have been more precise if collected at the county or local level. However, we believe that the former levels are valid, because the state level should be a good analytic unit in terms of motivation for the treatment (i.e., the drug reformulation policy). For a nationwide policy, exploring state-level outcomes can be more accurate than county- or individual-level outcomes; it would be difficult to measure the proportion of a state that is affected by a nationwide policy at the individual level.

Another limitation is that OxyContin misuse rates were self-reported and collected by a questionnaire regarding individuals’ misuse of OxyContin. As a result, the rates may be underreported, as respondents might hesitate to disclose their misuse of illegal drugs. But as Mackeiee-Liu argued in her paper, any measurement error of misuse rates might not bias estimates, since it might not vary by state (Mackenzie-Liu, 2021).

A further limitation concerns the DID model itself. The DID model has some inherent limitations, and our analysis might not reveal the causal effects of the reformulation. For example, concurrent policies might affect drug abuse violations and motor vehicle theft. Even though we have controlled for a variety of economic conditions and state policies, we cannot guarantee that no other factors will bias the results.

In addition, the full trend-break DID model found no significant effects on arrest rates for drug offenses. Beyond drug possession and sales, drug abuse violations include the manufacture, distribution, and transportation of illegal drugs and offenses related to illegal activities such as involvement with other offenders or illicit markets (Abuse, 2021). It is likely that analysis of other types of drug-related offenses might yield different results. Due to data limitations, the study was only able to examine the effects on drug offenses, including possession and sales. Future research could explore the reformulation’s effects on other types of drug-related offenses.

## 8 Conclusion

The study suggests that the OxyContin reformulation increases arrest rates for drug abuse violations and motor vehicle theft. The unintended consequences of the OxyContin reformulation have contributed to a growing body of literature on the negative effects of drug reformulation (Alpert et al., 2018; Beheshti, 2019; Evans et al., 2019; Abuse, 2021). Many healthcare issues and the high costs of the U.S. criminal justice system will accompany implementation of the reformulation. Who will be held responsible for these negative consequences? If the government is responsible, what funding source will cover the costs imposed by the reformulation? If the government uses other funding sources to solve these issues, will that trigger new problems? These questions should be addressed in future research.

Given this study’s finding that the OxyContin reformulation, which was designed to reduce the opioid crisis, instead increased arrest rates for drug abuse violations and rates for motor vehicle theft, innovative public policy will be required. Effective approaches must be more thoughtful about the various needs of opioid users and potential negative externalities following the implementation of new policies.

Further, the OxyContin reformulation is another example of drug policy that requires improvement, falling in line with the likes of the Mandatory Minimum Sentencing (Caulkins et al., 1997; Bjerk, 2017) and Marijuana Laws, which fail to address the underlying causes of drug use and related harms. As such, US drug policies should be thoroughly re-evaluated, placing greater emphasis on addressing the root causes of drug trade and abuse. Policymakers should reflect on the impact of their interventions and consider strategies more comprehensive.

## Data Availability

Publicly available datasets were analyzed in this study. This data can be found here: 1. NSDUH (National Survey on Drug Use and Health): https://www.datafiles.samhsa.gov 2. Crime Data Explorer: https://cde.ucr.cjis.gov/LATEST/webapp/#/pages/explorer/crime/arrest 3. Children’s Bureau Child Maltreatment Reports: https://acf.gov/cb/data-research/child-maltreatment 4. U.S. Census Bureau: https://data.census.gov/.

## References

[B1] Abuse N. I. on D. (2018). Heroin use is driven by its low cost and high availability. United States: National Institute on Drug Abuse. Available online at: https://nida.nih.gov/publications/research-reports/prescription-opioids-heroin/heroin-use-driven-by-its-low-cost-high-availability (Accessed January 10, 2026).

[B2] Abuse N. I. on D. (2021). Introduction. United States: National Institute on Drug Abuse website. Available online at: https://nida.nih.gov/publications/principles-drug-abuse-treatment-criminal-justice-populations-research-based-guide/introduction (Accessed March 15, 2022).

[B3] Alpert A. Powell D. Pacula R. L. (2018). Supply-side drug policy in the presence of substitutes: evidence from the introduction of abuse-deterrent opioids. Am. Econ. J. Econ. Policy 10 (4), 1–35. 10.1257/pol.20170082 34326924 PMC8317673

[B4] Anglin M. D. Speckart G. (1988). Narcotics use and crime: a multisample, multimethod analysis. Criminology 26 (2), 197–233. 10.1111/j.1745-9125.1988.tb00839.x

[B5] Arrests Offense Counts (2019). “Federal Bureau of Investigation,” in Crime data explorer. Available online at: https://crime-data-explorer.app.cloud.gov/pages/explorer/crime/arrest (Accessed March 15, 2022).

[B6] Automation of reports and consolidated order System user manual. (2023). Available online at: https://apps2.deadiversion.usdoj.gov/manuals/arcos_online_user_manual.pdf (Accessed June 23, 2022).

[B7] Barton W. I. (1980). Drug histories and criminality: survey of inmates of state correctional facilities, January 1974. Int. Journal Addict. 15 (2), 233–258. 10.3109/10826088009040011 7399755

[B8] Beheshti D. (2019). Adverse health effects of abuse‐deterrent opioids: evidence from the reformulation of OxyContin. Health Economics 28 (12), 1449–1461. 10.1002/hec.3944 31715653

[B9] Bjerk D. (2017). Mandatory minimums and the sentencing of federal drug crimes. J. Leg. Stud. 46 (1), 93–128. 10.1086/690205

[B10] Butler S. F. Cassidy T. A. Chilcoat H. Black R. A. Landau C. Budman S. H. (2013). Abuse rates and routes of administration of reformulated extended-release oxycodone: initial findings from a sentinel surveillance sample of individuals assessed for substance abuse treatment. J. Pain 14 (4), 351–358. 10.1016/j.jpain.2012.08.008 23127293

[B11] Card D. (1992). Using regional variation in wages to measure the effects of the federal minimum wage. Ilr Rev. 46 (1), 22–37. 10.1177/001979399204600103

[B12] Case A. Deaton A. (2015). Rising morbidity and mortality in midlife among white Non-Hispanic Americans in the 21st century. Proc. Natl. Acad. Sci. 112 (49), 15078–15083. 10.1073/pnas.1518393112 26575631 PMC4679063

[B13] Caulkins J. P. Rydell C. P. Schwabe W. L. Chiesa J. (1997). Mandatory minimum drug sentences: throwing away the key or the taxpayers' money? . Santa Monica, CA: RAND Corporation.

[B14] Child Maltreatment (2017). “U.S. Department of Health & Human Services, administration for children and families, administration on children, youth and families, children’s,” in Child Maltreatment 2015. Available online at: http://www.acf.hhs.gov/programs/cb/research-data-technology/statistics-research/child-maltreatment (Accessed June 23, 2022).

[B15] Child Maltreatment (2021). “U.S. Department of Health & Human Services, administration for children and families, administration on children, youth and families, children’s bureau,” in Child Maltreatment 2019. Available online at: https://www.acf.hhs.gov/cb/research-data-technology/statistics-research/child-maltreatment (Accessed June 23, 2022).

[B16] Children's Bureau (2020). Child Maltreatment 2019. Washington, DC: U.S. Department of Health & Human Services, Administration for Children and Families. Available online at: https://www.acf.hhs.gov/cb/research-data-technology/statistics-research/child-maltreatment (Accessed June 23, 2022).

[B17] Cicero T. J. Ellis M. S. Surratt H. L. (2012). Effect of abuse-deterrent formulation of OxyContin. N. Engl. J. Med. 367 (2), 187–189. 10.1056/NEJMc1204141 22784140

[B18] Cozzi M. (2007). “Hard drugs addiction, drug violations and property crimes in the US,” in *2007 meeting papers* (No. 51) (Minneapolis: Society for Economic Dynamics).

[B19] Drug Enforcement Administration (DEA) (2022). Diversion control division, Drug & Chemical Evaluation Section March 2020, “Oxycodone.”. Available online at: https://www.deadiversion.usdoj.gov/drug_chem_info/oxycodone/oxycodone.pdf (Accessed June 23, 2022).

[B20] Evans W. N. Lieber E. M. Power P. (2019). How the reformulation of OxyContin ignited the heroin epidemic. Rev. Econ. Statistics 101 (1), 1–15. 10.1162/rest_a_00755

[B21] Flood S. King M. Rodgers R. Ruggles S. Rom,jklbert Warren J. Westberry M. (2022). “Integrated public use Microdata Series, Current Population Survey,”. Minneapolis, MN: IPUMS, University of Minnesota. 10.18128/D030.V10.0

[B22] Gladden R. M. Martinez P. Seth P. (2016). Fentanyl law enforcement submissions and increases in synthetic opioid–involved overdose deaths—27 states, 2013–2014. MMWR. Morb. Mortality Weekly Report 65, 837–843. 10.15585/mmwr.mm6533a2 27560775

[B23] Harrison L. Gfroerer J. (1992). The intersection of drug use and criminal behavior: results from the National Household Survey on Drug Abuse. Crime & Delinquency 38 (4), 422–443. 10.1177/0011128792038004002

[B24] Hedegaard H. Miniño A. M. Warner M. (2020). “Drug overdose deaths in the United States, 1999-2018,”. Hyattsville, MD: National Center for Health Statistics (NCHS), Centers for Disease Control and Prevention. 356, 1–8. 10.15620/cdc:112340

[B25] Johnson B. D. Goldstein P. J. Preble E. Schmeidler J. Lipton D. S. Spunt B. (1985). *Taking care of business: the economics of crime by heroin abusers*. Lexington, MA: DC Heath.

[B26] Kolodny A. Courtwright D. T. Hwang C. S. Kreiner P. Eadie J. L. Clark T. W. (2015). The prescription opioid and heroin crisis: a public health approach to an epidemic of addiction. Annu. Review Public Health 36 (1), 559–574. 10.1146/annurev-publhealth-031914-122957 25581144

[B27] Mackenzie-Liu M. (2021). From fostering hope to lingering harm: the unintended impact of the OxyContin reformulation on child welfare utilization. Soc. Serv. Rev. 95 (1), 36–65. 10.1086/713378

[B47] Maclean J. C. Mallatt J. Ruhm C. J. Simon K. (2021). “Economic studies on the opioid crisis: costs, causes, and policy responses,” in Oxford research encyclopedia of economics and finance.

[B48] Maclean J. C. Mallatt J. Ruhm C. J. Simon K. (2022). The opioid crisis, health, healthcare, and crime: a review of quasi-experimental economic studies. ANNALS American Academy Politic. Soc. Sci. 703 (1), 15–49.

[B28] Makary M. A. Overton H. N. Wang P. (2017). Overprescribing is major contributor to opioid crisis. Bmj 359, j4792. 10.1136/bmj.j4792 29051174

[B29] Manchikanti L. Fellows S. H. B. Janata J. W. Pampati V. Grider J. S. Boswell M. V. (2012). Opioid epidemic in the United States. Pain Physician 15 (3S), ES9–E38. 22786464

[B30] National Survey on Drug (2013). National Survey on Drug use and health: Model-Based prevalence estimates (50 States and the District of Columbia). Available online at: https://www.samhsa.gov/data/sites/default/files/NSDUHsaePercents2014.pdf. (Accessed June 23, 2022)

[B31] Nurco D. N. Hanlon T. E. Kinlock T. W. Duszynski K. R. (1988). Differential criminal patterns of narcotic addicts over an addiction career. Criminology 26 (3), 407–423. 10.1111/j.1745-9125.1988.tb00848.x

[B32] O’Donnell J. K. Gladden R. M. Seth P. (2017a). Trends in deaths involving heroin and synthetic opioids excluding methadone, and law enforcement drug product reports, by census region—United States, 2006–2015. MMWR. Morb. Mortality Weekly Report 66, 897–903. 10.15585/mmwr.mm6634a2 PMC5657786 28859052

[B33] O’Donnell J. K. Halpin J. Mattson C. L. Goldberger B. A. Gladden R. M. (2017b). Deaths involving fentanyl, fentanyl analogs, and U-47700—10 states, July–December 2016. MMWR. Morb. Mortality Weekly Report 66, 1197–1202. 10.15585/mmwr.mm6643e1 PMC5689219 29095804

[B34] Park S. (2021). *Three Essays on the Broader Effects of the Opioid Crisis* (Doctoral dissertation, RAND).

[B35] Paulozzi L. J. (2012). Prescription drug overdoses: a review. J. Safety Research 43 (4), 283–289. 10.1016/j.jsr.2012.08.009 23127678

[B36] Rosenbaum P. R. Rosenbaum P. (2010). “Design of observational studies,”, 10. New York: Springer, 978.

[B37] Rosenfeld R. Gaston S. Spivak H. Irazola S. (2017). Assessing and responding to the recent homicide rise in the United States. CrimRxiv, University of Manchester.

[B38] Rudd R. A. Paulozzi L. J. Bauer M. J. Burleson R. W. Carlson R. E. Dao D. (2014). Increases in heroin overdose deaths—28 states, 2010 to 2012. MMWR Morb. Mortal. Wkly. Rep. 63 (39), 849–854. 25275328 PMC4584873

[B39] Rudd R. A. Aleshire N. Zibbell J. E. Gladden R. M. (2016). Increases in drug and opioid overdose deaths—United States, 2000–2014. Am. J. Transplant. 16 (4), 1323–1327. 10.1111/ajt.13776 26720857

[B40] Ruggles S. Flood S. Foster S. Goeken R. Pacas J. Schouweiler M. (2021). Minneapolis, MN: IPUMS. 10.18128/D010.V11.0

[B41] Rummans T. A. Burton M. C. Dawson N. L. (2018). “How good intentions contributed to bad outcomes: the opioid crisis,”, 93. Elsevier, 344–350. 10.1016/j.mayocp.2017.12.020 29502564

[B42] Stevens J. P. Wall M. J. Novack L. Marshall J. Hsu D. J. Howell M. D. (2017). The critical care crisis of opioid overdoses in the United States. Ann. Am. Thorac. Soc. 14 (12), 1803–1809. 10.1513/AnnalsATS.201701-022OC 28800256 PMC5802515

[B43] Szalavitz M. Rigg K. K. (2017). The curious (dis) connection between the opioid epidemic and crime. Subst. Use & Misuse 52 (14), 1927–1931. 10.1080/10826084.2017.1376685 28952839

[B44] U.S. Department of Justice (2004). “U.S. Department of Justice, Federal Bureau of Investigation,” in Uniform crime reporting handbook, 143. Available online at: http://www2.fbi.gov/ucr/handbook/ucrhandbook04.pdf (Accessed March 29, 2022).

[B45] Wolff C. Dowd W. N. Ali M. M. McClellan C. Meinhofer A. Glos L. (2020). The impact of the abuse-deterrent reformulation of extended-release OxyContin on prescription pain reliever misuse and heroin initiation. Addict. Behaviors 105, 106268. 10.1016/j.addbeh.2019.106268 32036188

[B46] Yamamoto M. Ran W. (2013). Drug abuse violations in communities: community newspapers as a macro-level source of social control. Journalism & Mass Commun. Q. 90 (4), 629–651. 10.1177/1077699013503164

