# Supplementary material for: OxyContin reformulation and drug-related-arrest rates, property-related crimes, child maltreatment and food pantry participation
Source: Front Pharmacol. 2026 Mar 27;17:1681241. doi: 10.3389/fphar.2026.1681241 (PMC13066133; doi:10.3389/fphar.2026.1681241)
Supplement: Supplementary file 1 [file Table1.docx]

**Appendix A**

**Table A1**. Effect of OxyContin Reformulation on the Outcomes, Dichotomous Treatment Model

|  | Low OxyContin misuse vs High OxyContin misuse | | | Low OxyContin misuse vs Medium OxyContin misuse | | |
| --- | --- | --- | --- | --- | --- | --- |
|  | (1) | (2) | **Preferred Model**  (3) | (4) | (5) | **Preferred Model**  (6) |
| Rates for Property Crime per 100,000 | 96.29 | 90.09 | 39.52 | -124.39 | -62.87 | -94.66 |
|  | (139.41) | (102.85) | (86.00) | (169.65) | (110.77) | (78.44) |
| R^2^ | 0.930 | 0.945 | 0.954 | 0.928 | 0.950 | 0.959 |
| N | 646 | 646 | 628 | 646 | 646 | 622 |
| Number of States + DC | 34 | 34 | 34 | 34 | 34 | 34 |
| State and Time-varying Covariates | No | Yes | Yes | No | Yes | Yes |
| Policy Variables | No | No | Yes | No | No | Yes |
| Rates for Burglary per 100,000 | 26.85 | 23.33 | 17.63 | -49.09 | -37.68 | -39.19 |
|  | (40.79) | (26.71) | (19.11) | (54.48) | (32.82) | (25.88) |
| R^2^ | 0.933 | 0.951 | 0.960 | 0.926 | 0.951 | 0.958 |
| N | 646 | 646 | 628 | 646 | 646 | 622 |
| Number of States + DC | 34 | 34 | 34 | 34 | 34 | 34 |
| State and Time-varying Covariates | No | Yes | Yes | No | Yes | Yes |
| Policy Variables | No | No | Yes | No | No | Yes |
| Rates for Larceny per 100,000 | 16.23 | 39.13 | 4.48 | -68.31 | -30.65 | -51.31 |
|  | (101.70) | (65.93) | (62.20) | (107.91) | (70.34) | (51.98) |
| R^2^ | 0.911 | 0.936 | 0.945 | 0.921 | 0.947 | 0.955 |
| N | 646 | 646 | 628 | 646 | 646 | 622 |
| Number of States + DC | 34 | 34 | 34 | 34 | 34 | 34 |
| State and Time-varying Covariates | No | Yes | Yes | No | Yes | Yes |
| Policy Variables | No | No | Yes | No | No | Yes |
| Child Victimization per 1,000 Children | -0.01 | -0.15 | 0.35 | -2.63 | -1.99 | -1.39 |
|  | (1.08) | (1.20) | (1.22) | (2.46) | (1.66) | (1.61) |
| R^2^ | 0.781 | 0.811 | 0.818 | 0.686 | 0.789 | 0.802 |
| N | 641 | 641 | 623 | 640 | 640 | 616 |
| Number of States + DC | 34 | 34 | 34 | 34 | 34 | 34 |
| State and Time-varying Covariates | No | Yes | Yes | No | Yes | Yes |
| Policy Variables | No | No | Yes | No | No | Yes |
| Food Pantry Participation Rate | -0.09 | -0.14 | -0.12 | 0.09 | 0.02 | 0.13 |
|  | (0.24) | (0.19) | (0.24) | (0.15) | (0.16) | (0.19) |
| R^2^ | 0.596 | 0.639 | 0.647 | 0.662 | 0.682 | 0.685 |
| N | 646 | 646 | 628 | 646 | 646 | 622 |
| Number of States + DC | 34 | 34 | 34 | 34 | 34 | 34 |
| State and Time-varying Covariates | No | Yes | Yes | No | Yes | Yes |
| Policy Variables | No | No | Yes | No | No | Yes |

*Notes:* *** p<0.01, ** p<0.05, * p<0.1. Figures in parentheses are the respective robust standard errors, clustered at state level. N reports state-year observations. The dichotomous treatment is defined based on the tertiles of OxyContin misuse at the state level prior to the reformulation. States are categorized into 'Low', 'Medium', and 'High' OxyContin misuse groups. State fixed effects and year fixed effects are included in all specifications. State and time-varying covariates include log population, population per square mile, share non-Hispanic Black, share Hispanic, share with high school degree, share with less than high school degree, share with multiple age groups (0-19, 20-39, 65+), poverty rate, and unemployment rate. We also account for state policy variables including indicators for prescription drug monitoring programs, pill mill legislation, medical marijuana laws, and active and legal medical marijuana dispensaries. Regressions are weighted by population. Years 2001–2019 are used.
