# Supplementary material for: OxyContin reformulation and drug-related-arrest rates, property-related crimes, child maltreatment and food pantry participation
Source: Front Pharmacol. 2026 Mar 27;17:1681241. doi: 10.3389/fphar.2026.1681241 (PMC13066133; doi:10.3389/fphar.2026.1681241)
Supplement: Supplementary file 2 [file Table2.docx]

**Appendix A**

**Table A2**. Effect of OxyContin reformulation on state arrests for arrests for drug offenses rates, and rates for property crimes, burglary, larceny, child victimization, and food pantry participation rate, trend-break model

|  | Arrest Rates for Drug Offenses per 100,000 | | | | Rates for Property Crime per 100,000 | | | |
| --- | --- | --- | --- | --- | --- | --- | --- | --- |
|  | (5) | (6) | (7) | **Preferred Model**  (8) | (9) | (10) | (11) | **Preferred Model**  (12) |
| 1-year effect | 100.91 | 81.35 | **112.33*** | 86.16 | 154.98 | 124.49 | **210.73*** | 64.75 |
|  | (85.20) | (67.71) | (66.37) | (66.32) | (125.76) | (131.85) | (125.49) | (168.41) |
| 2-year effect | 88.40 | 82.17 | **119.03*** | 87.72 | 91.22 | 67.25 | 179.32 | 5.13 |
|  | (79.48) | (68.75) | (60.26) | (68.80) | (168.14) | (173.49) | (136.82) | (201.79) |
| 3-year effect | 75.88 | 82.99 | **125.74**** | 89.27 | 27.46 | 10.01 | 147.91 | -54.50 |
|  | (76.26) | (73.95) | (55.59) | (75.30) | (214.47) | (220.37) | (152.44) | (238.56) |
| 4-year effect | 63.37 | 83.81 | **132.44**** | 90.83 | -36.30 | -47.23 | 116.50 | -114.13 |
|  | (75.86) | (82.54) | (52.73) | (84.90) | (262.66) | (269.78) | (171.17) | (277.38) |
| 5-year effect | 50.86 | 84.63 | **139.14***** | 92.38 | -100.06 | -104.47 | 85.10 | -173.76 |
|  | (78.32) | (93.58) | (51.99) | (96.68) | (311.84) | (320.54) | (192.10) | (317.50) |
| 6-year effect | 38.34 | 85.44 | **145.85***** | 93.93 | -163.82 | -161.71 | 53.69 | -233.38 |
|  | (83.39) | (106.31) | (53.46) | (109.95) | (361.63) | (372.10) | (214.59) | (358.48) |
| 7-year effect | 25.83 | 86.26 | **152.55***** | 95.49 | -227.58 | -218.95 | 22.28 | -293.01 |
|  | (90.63) | (120.20) | (56.96) | (124.23) | (411.78) | (424.18) | (238.20) | (400.05) |
| 8-year effect | 13.31 | 87.08 | **159.26**** | 97.04 | -291.34 | -276.19 | -9.13 | -352.64 |
|  | (99.57) | (134.89) | (62.15) | (139.20) | (462.20) | (476.60) | (262.62) | (442.06) |
| 9-year effect | 0.80 | 87.90 | **165.96**** | 98.60 | -355.10 | -333.43 | -40.53 | -412.26 |
|  | (109.70) | (150.14) | (68.65) | (154.67) | (512.79) | (529.26) | (287.65) | (484.38) |
| R^2^ | 0.813 | 0.844 | 0.842 | 0.843 | 0.927 | 0.947 | 0.954 | 0.954 |
| N | 952 | 952 | 922 | 922 | 969 | 969 | 939 | 939 |
| Number of states + DC | 51 | 51 | 51 | 51 | 51 | 51 | 51 | 51 |
| State and time-varying covariates | No | Yes | Yes | Yes | No | Yes | Yes | Yes |
| Linear time trend interaction | Yes | Yes | No | Yes | Yes | Yes | No | Yes |
| Policy variables | No | No | Yes | Yes | No | No | Yes | Yes |

**Table A2**. Effect of OxyContin reformulation on state arrests for arrests for drug offenses rates, and rates for property crimes, burglary, larceny, child victimization, and food pantry participation rate, trend-break model (continued)

|  | Rates for Burglary per 100,000 | | | | Rates for Larceny per 100,000 | | | |
| --- | --- | --- | --- | --- | --- | --- | --- | --- |
|  | (13) | (14) | (15) | **Preferred Model**  (16) | (17) | (18) | (19) | **Preferred Model**  (20) |
| 1-year effect | 38.97 | 34.34 | 36.26 | 6.11 | 104.76 | 91.14 | **197.63**** | 69.46 |
|  | (52.64) | (48.67) | (35.91) | (62.79) | (77.84) | (74.96) | (80.71) | (93.98) |
| 2-year effect | 30.13 | 22.75 | 31.56 | -4.42 | 40.07 | 25.57 | **152.47*** | -0.49 |
|  | (66.08) | (64.38) | (38.51) | (78.28) | (109.05) | (104.79) | (89.76) | (115.88) |
| 3-year effect | 21.28 | 11.17 | 26.86 | -14.95 | -24.63 | -40.00 | 107.30 | -70.43 |
|  | (80.57) | (81.49) | (43.35) | (94.87) | (142.77) | (138.60) | (102.70) | (140.94) |
| 4-year effect | 12.44 | -0.42 | 22.15 | -25.48 | -89.31 | -105.57 | 62.13 | -140.38 |
|  | (95.62) | (99.27) | (49.77) | (112.07) | (177.56) | (174.08) | (118.26) | (167.75) |
| 5-year effect | 3.60 | -12.01 | 17.45 | -36.01 | -154.01 | -171.14 | 16.96 | -210.33 |
|  | (111.02) | (117.41) | (57.25) | (129.64) | (212.92) | (210.40) | (135.54) | (195.58) |
| 6-year effect | -5.25 | -23.59 | 12.75 | -46.54 | -218.70 | -236.71 | -28.21 | -280.27 |
|  | (126.62) | (135.78) | (65.42) | (147.44) | (248.59) | (247.18) | (153.96) | (224.07) |
| 7-year effect | -14.09 | -35.18 | 8.05 | -57.07 | -283.39 | -302.28 | -73.37 | -350.22 |
|  | (142.38) | (154.30) | (74.05) | (165.41) | (284.45) | (284.25) | (173.16) | (252.98) |
| 8-year effect | -22.93 | -46.77 | 3.34 | -67.61 | -348.08 | -367.85 | -118.54 | -420.16 |
|  | (158.23) | (172.90) | (83.01) | (183.49) | (320.45) | (321.50) | (192.91) | (282.19) |
| 9-year effect | -31.78 | -58.35 | -1.36 | -78.14 | -412.78 | -433.42 | -163.71 | -490.11 |
|  | (174.16) | (191.58) | (92.19) | (201.65) | (356.54) | (358.88) | (213.04) | (311.61) |
| R^2^ | 0.926 | 0.949 | 0.956 | 0.956 | 0.921 | 0.943 | 0.949 | 0.949 |
| N | 969 | 969 | 939 | 939 | 969 | 969 | 939 | 939 |
| Number of states + DC | 51 | 51 | 51 | 51 | 51 | 51 | 51 | 51 |
| State and time-varying covariates | No | Yes | Yes | Yes | No | Yes | Yes | Yes |
| Linear time trend interaction | Yes | Yes | No | Yes | Yes | Yes | No | Yes |
| Policy variables | No | No | Yes | Yes | No | No | Yes | Yes |

**Table A2**. Effect of OxyContin reformulation on state arrests for arrests for drug offenses rates, and rates for property crimes, burglary, larceny, child victimization, and food pantry participation rate, trend-break model (continued)

|  | Child Victimization per 1,000 Children | | | | Food Pantry Participation Rate | | | |
| --- | --- | --- | --- | --- | --- | --- | --- | --- |
|  | (25) | (26) | (27) | **Preferred Model**  (28) | (29) | (30) | (31) | **Preferred Model**  (32) |
| 1-year effect | -0.70 | -1.02 | -4.69 | -2.44 | -0.19 | -0.20 | -0.21 | -.15 |
|  | (2.65) | (2.81) | (2. 80) | (2.72) | (0.60) | (0.59) | (0.51) | (0.65) |
| 2-year effect | 0.22 | 0.17 | -3.87 | -1.19 | -0.19 | -0.23 | -0.25 | -.18 |
|  | (2.94) | (3.03) | (2.70) | (3.11) | (0.58) | (0.60) | (0.45) | (0.67) |
| 3-year effect | 1.13 | 1.36 | -3.06 | 0.05 | -0.20 | -0.26 | -0.29 | -0.20 |
|  | (3.29) | (3.38) | (2.62) | (3.61) | (0.60) | (0.63) | (0.40) | (0.70) |
| 4-year effect | 2.04 | 2.55 | -2.24 | 1.30 | -0.20 | **-**0.28 | -0.33 | -0.23 |
|  | (3.68) | (3.81) | (2.56) | (4.18) | (0.65) | (0.68) | (0.36) | (0.75) |
| 5-year effect | 2.96 | 3.74 | -1.43 | 2.55 | -0.21 | **-**0.31 | -0.36 | -0.26 |
|  | (4.10) | (4.31) | (2.52) | (4.79) | (0.73) | (0.74) | (0.34) | (0.81) |
| 6-year effect | 3.87 | 4.93 | -0.62 | 3.80 | -0.21 | **-**0.34 | -0.40 | **-**0.28 |
|  | (4.54) | (4.84) | (2.52) | (5.44) | (0.82) | (0.82) | (0.34) | (0.89) |
| 7-year effect | 4.78 | 5.12 | 0.20 | 5.05 | -0.22 | **-**0.37 | -0.44 | **-**0.31 |
|  | (4.99) | (5.40) | (2.54) | (6.10) | (0.93) | (0.91) | (0.35) | (0.97) |
| 8-year effect | 5.70 | 7.31 | 1.01 | 6.29 | -0.23 | **-**0.39 | -0.48 | **-**0.33 |
|  | (5.46) | (5.99) | (2.58) | (6.78) | (1.05) | (1.00) | (0.39) | (1.06) |
| 9-year effect | 6.61 | 8.50 | 1.83 | 7.54 | -0.23 | **-**0.42 | -0.52 | **-**0.36 |
|  | (5.94) | (6.59) | (2.65) | (7.47) | (1.17) | (1.10) | (0.43) | (1.15) |
| R^2^ | 0.710 | 0.778 | 0.791 | 0.792 | 0.630 | 0.660 | 0.663 | 0.663 |
| N | 962 | 962 | 932 | 932 | 969 | 969 | 939 | 939 |
| Number of states + DC | 51 | 51 | 51 | 51 | 51 | 51 | 51 | 51 |
| State and time-varying covariates | No | Yes | Yes | Yes | No | Yes | Yes | Yes |
| Linear time trend interaction | Yes | Yes | No | Yes | Yes | Yes | No | Yes |
| Policy variables | No | No | Yes | Yes | No | No | Yes | Yes |

*Notes:* *** p<0.01, ** p<0.05, * p<0.1. Figures in parentheses are the respective robust standard errors, clustered at state level. N reports state-year observations. Except for models (3), (7), (11), (15), (19) and (23), each model also includes a post indicator interacted with initial OxyContin misuse, a linear time trend interacted with initial OxyContin misuse, and a post-2011 linear trend interacted with initial OxyContin misuse. State fixed effects and year fixed effects are included in all specifications. State and time-varying covariates include log population, population per square mile, share non-Hispanic Black, share Hispanic, share with high school degree, share with less than high school degree, share with multiple age groups (0-19, 20-39, 65+), poverty rate, and unemployment rate. We also account for state policy variables including indicators for prescription drug monitoring programs, pill mill legislation, medical marijuana laws, and active and legal medical marijuana dispensaries. Regressions are weighted by population. Years 2001–2019 are used.
